# The FRAXA and FRAXE allele repeat size of boys from the Avon Longitudinal Study of Parents and Children (ALSPAC)

**DOI:** 10.12688/wellcomeopenres.15342.2

**Published:** 2020-01-27

**Authors:** Rosie Clark, Steven Gregory, Susan Ring, Patricia Jacobs, Sarah Ennis, Anna Murray, Genette Ellis, Jean Golding, Kate Northstone, Marcus Pembrey

**Affiliations:** 1 Population Health Sciences, Bristol Medical School, University of Bristol, Oakfield House, Oakfield Grove, Bristol, BS8 2BN, UK; 2 MRC Integrative Epidemiology Unit, University of Bristol, Oakfield House, Oakfield Grove, Bristol, BS8 2BN, UK; 3 Human Genetics, Faculty of Medicine, University of Southampton, University Road, Southampton, SO17 1BJ, UK; 4 Medical School, University of Exeter, Barrack Road, Exeter, EX2 5DW, UK

**Keywords:** ALSPAC, longitudinal cohort, FRAXA, FRAXE, Triplet repeats

## Abstract

The FRAXA and FRAXE alleles of the FMR1 and FMR2 genes located on the X chromosome contain varying numbers of trinucleotide repeats. Large numbers of repeats at FRAXA (full mutations) manifest as Fragile X syndrome, associated with mental impairment that affects males more severely. In this paper, we present the dataset of frequencies of FRAXA and FRAXE repeat size extracted from DNA samples collected from boys enrolled in the Avon Longitudinal Study of Parents and Children (ALSPAC). DNA data were extracted from samples collected in ALSPAC clinics from several types of samples: cord blood, venepuncture blood taken at 43 months, 61 months, seven years or nine years. The DNA was amplified at FRAXA and FRAXE using fluorescent PCR in the Wessex Regional Genetics Laboratory, Salisbury District Hospital. The mean repeat size for FRAXA is 28.92 (S.D. 5.44), the median 30 and the range 8 to 68. There were particularly high numbers of boys with repeat sizes of 20 (10.67%) and 23 (7.35%). The mean repeat size for FRAXE is 17.41 (S.D. 3.94), with median of 16 and range of 0 to 61. There is a relatively high degree of variation of the FRAXA repeat size particularly and we suggest the extensive data available from the ALSPAC study opens up areas of research into understanding phenotypes associated with relatively unexplored repeat sizes. This could be particularly interesting for the lower repeat sizes occurring with high frequency at FRAXA in this population. As the data can be linked to exposures and phenotypes, it will provide a resource for researchers worldwide.

## Introduction

The mutation associated with the Fragile X syndrome is within a CGG trinucleotide repeat array in the FMR1 gene, identified in 1991 ( Pieretti *et al*., 1991). The gene is located in the Xq27.3 region of the long arm of the X chromosome ( Lubs, 1969; Sutherland, 1979; Sutherland, 1977). The array contains a variable number of repeats, and each repeat size is regarded in genetic terms as an FMR1 allele (often referred to as a FRAXA allele) (reviewed in Pembrey *et al*., 2001). Similarly, FRAXE syndrome is caused by the expansion of a GCC repeat in the FMR2 gene ( Knight *et al*., 1993). FRAXA allele classes are categorised by the number of repeats and defined as full mutation (>200), premutation (61–200), intermediate (41–60), common (11–40) and minimal (<11) ( Murray *et al*., 1996). FRAXE allele classes are similarly defined, but are slightly different for intermediate (31–60) and common (11–31) classes.

Full mutations of FRAXA (> 200 CGG repeats with silencing of the FMR1 gene) are more common than those of FRAXE ( Knight *et al*., 1996); in the literature, the population prevalence of full FRAXA mutations in males was estimated to be between 1 in 4000 and 1 in 5000, though acknowledging biases due to informed consent ( Chandrasekara *et al*., 2017; Youings *et al*., 2000). For females the estimation is lower, at around 1 in 8000 ( Crawford *et al*., 1999). FRAXE full mutation prevalence in males has been estimated at 1 in 23,400 ( Youings *et al*., 2000).

Individuals with a full mutation inherit it from a female who carries a full mutation or premutation ( Pembrey *et al*., 2001). Around a third of female gene carriers are intellectually impaired, usually less severely than males ( Pembrey *et al*., 2001). Most males with Fragile X syndrome (full mutation) have a severe to moderate degree of mental impairment ( Pembrey *et al*., 2001). The full mutation type for FRAXE syndrome has been found to result in much milder impairment than the FRAXA phenotype ( Crawford *et al*., 1999). 

In females, there is a well-established phenotypic effect of FRAXA premutation sized alleles of an increased risk of premature ovarian failure ( Chandrasekara *et al*., 2017; Macpherson *et al*., 2003; Youings *et al*., 2000). There is also some evidence of an increased incidence of late-onset neurological disorder characterised by tremor and ataxia (FXTAS) in premutation males, with around 40% at risk, and, to a lesser extent, females, at ~8% ( Chandrasekara *et al*., 2017; Hagerman *et al*., 2001; Hagerman *et al*., 2004). Several studies have suggested that intermediate alleles at both FRAXE and FRAXA may have a role in cognitive impairment in boys, more reliably for FRAXA ( Murray *et al*., 1996; Youings *et al*., 2000). However, this finding has not been replicated in some other multinational studies ( Youings *et al*., 2000). Specifically, a study investigating FRAXA intermediate alleles in ALSPAC data even found a significant deficiency of special educational needs (SEN) cases in boys with FRAXA intermediate expansions ( Ennis *et al*., 2006).

An issue within the literature is that the definitions of the boundaries of classes of repeat numbers are sometimes different across studies (e.g. changes over time due to knowledge base or differing clinical definitions), thus making it harder to compare frequencies (see Cornish *et al*., 2008; Pembrey *et al*., 2001; Youings *et al*., 2000). The general variation of frequencies of repeat sizes is often overlooked, as are phenotypic associations with relatively low repeat sizes (particularly differences within allele classes). Additionally, some studies have found a correlation between the length of repeats and degree of phenotype, even within allele classes ( Cornish *et al*., 2008).

Here we present a dataset of repeat size frequencies at FRAXA and FRAXE of boys from the ALSPAC study, which could provide an important resource for researchers internationally.

## Materials and methods

### Ethical approval and consent

Ethical approval for the study was obtained from the ALSPAC Ethics and Law Committee and the Local Research Ethics Committees ( Birmingham, 2018). Informed consent for the use of data via questionnaires and clinics was obtained from participants following the recommendations of the ALSPAC Ethics and Law Committee at the time. Questionnaires were completed by parents in their own homes and the return of a questionnaire to the study offices was interpreted as giving tacit consent to the study. Full details of the approvals obtained are available from the ethics pages of the study website ( http://www.bristol.ac.uk/alspac/researchers/research-ethics/). Study members have the right to withdraw their consent for elements of the study or from the study entirely at any time. Consent for biological samples was collected in accordance with the Human Tissue Act (2004).

### The ALSPAC sample

The ALSPAC study was designed to include all pregnant mothers with an expected date of delivery between 1 ^st^ April 1991 and 31 ^st^ December 1992 living in a specific area in the South West Regional Health Authority of England ( Boyd *et al*., 2013; Fraser *et al*., 2013). The number of enrolled pregnant mothers was 14541, which resulted in 13988 infants who survived to one year. When the oldest children were approximately seven years of age, an attempt was made to bolster the initial sample with eligible cases who had failed to join the study originally. Including these additional children results in 14,901 infants who survived to one year. Data collection largely consisted of self-completion questionnaires (principally based on psychosocial factors, physical environments and health) filled in by mothers, partners, children and teachers. The study methodology ( Golding *et al*., 2001), enrolment and response rates are given in detail on the study website ( http://bristol.ac.uk/alspac/index.html). Please note that the study website contains details of all the data that is available through a fully searchable data dictionary and variable search tool ( http://www.bristol.ac.uk/alspac/researchers/our-data/). The data presented here on maternal age, parity, parental education levels and socioeconomic status were obtained during pregnancy from maternal self-completion questionnaires. 

### Sample collection

The DNA of children in the study was gathered by the ALSPAC study team; samples were collected from 5275 males in total ( Ennis *et al*., 2006). Permission for DNA extraction was obtained as written consent from the study mothers during pregnancy (for cord blood), as well as in ALSPAC clinics for venepuncture blood taken at 43 months, 61 months, seven years and nine years. Some samples were extracted from buccal wash when the child did not consent to venepuncture. The ALSPAC study team double coded all samples for anonymity.

### DNA amplification and analysis

Cord blood samples were collected in heparin at birth and stored at -70°C for five to eight years before DNA extraction ( Jones *et al*., 2000). Blood samples from children at 43 or 61 months were stored for one month to two years before extraction ( Jones *et al*., 2000). Samples from children at seven and nine years were stored at -20°C for up to three weeks before DNA extraction ( Jones *et al*., 2000). The samples were provided to the laboratory as 250ng aliquots in 96 well plates with eight wells on each plate left empty for laboratory control, consisting of DNA with known CGG repeat number and water controls ( Ennis *et al*., 2006). When there was both a cord blood and clinic sample were available for the same boy, the clinic sample was used to maximise genotyping efficiency, as heparin can inhibit PCR, and to minimise maternal contamination issues ( Ennis *et al*., 2006). The DNA was amplified using fluorescent PCR (involving ﬂuorescently labelled oligonucleotide primers) across the CGG FRAXA repeat and GCC FRAXE repeat. This took place in the Wessex Regional Genetics Laboratory.

Two multiplex PCR reactions were carried out, the details of which are given elsewhere ( Jones *et al*., 2000; Murray *et al*., 1996; Youings *et al*., 2000). For both multiplex reactions, products were denatured and separated on a polyacrylamide urea gel and then run on an ABI 373A Stretch machine for eight hours at 40 W, 2500 V and 35 mA ( Murray *et al*., 1996). The gel data were analysed on 672 GENESCAN software (ABI/Perkin Elmer) and then imported into GENOTYPER software (ABI/Perkin Elmer) to assign alleles ( Murray *et al*., 1996).

### Data processing

Genotype data were returned to the ALSPAC Study (University of Bristol). The anonymised dataset is in SPSS format and analysis was carried out using a combination of SPSS (version 11) statistical software and SAS (SAS Institute Inc., Cary, N.C.). The dataset includes: the ALSPAC pregnancy identifier (anonymised), whether the pregnancy was singleton/twins/triplets, the category of FRAXA/E repeat size, and the number of repeats at FRAXA/E.

## Dataset

In all, there were 5087 children for whom the number of repeats (RPTs) at FRAXA and 5070 at FRAXE were assessed. Of these, 192 were additional cases recruited at seven years old. The proportion of the 7676 boys in the study population for whom RPT data were obtained was 66.3% for FRAXA and 66.1% for FRAXE. Those who were missed included: those for whom permission had not been given and thus DNA was not available (2401); those where the assay had not worked (164), including those with high levels of premutations and the full mutation; and additionally, where there was not enough DNA, or samples had a heterozygous genotype indicating the possibility of two X chromosomes or maternal contamination (27) ( Ennis *et al*., 2006). As PCR is not possible for amplification of large premutations/full mutations, these individuals are not included in this dataset.

Table 1 shows the percentage of boys for whom there is FRAXA repeat data within each category of sociodemographic attributes. Among the parents of boys in the ALSPAC study, FRAXA repeat information was slightly more likely to be available when the education levels achieved by the mother and the father were higher, the father’s occupation was of a higher social class, and the mothers were older. However, there was no difference in availability by the number of pregnancies the mother had had where the baby reached viability (parity). The patterns in regard to FRAXE were similar.

**Table 1.** Proportion (and percentage) of boys for whom the number of FRAXA repeats (RPTs) have been obtained by sociodemographic attributes of their parents. The education level ranges from less than O-level/GCSE (A) to degree level qualification (E). The social class categories are as previously defined ( ‘Standard occupation classification’, 1990).

Variable	Proportion with FRAXA RPTs	% with FRAXA RPTs
*Maternal education*		
**A (lowest)**	755 /1322	57.1
**B**	418 /641	65.2
**C**	1577 /2237	70.5
**D**	1139 /1439	79.2
**E (highest)**	618 /812	76.1
*Paternal education*		
**A (lowest)**	974 /1612	60.4
**B**	343 /515	66.6
**C**	950 /1334	71.2
**D**	1129 /1579	71.5
**E (highest)**	863 /1143	75.5
*Social class (father’s* *Occupation*		
**I (Higher professional)**	479 /630	76.0
**II**	1373 /1916	71.7
**IIINm**	436 /619	70.4
**IIIM**	1187 /1779	66.7
**IV**	361 /535	67.5
**V (unskilled)**	110 /163	67.5
*Maternal age*		
**<20**	151 /327	46.2
**20–24**	778 /1378	56.5
**25–29**	1892 /2813	67.3
**30–34**	1397 /1998	69.9
**35+**	547 /766	71.4
*Parity of the mother*		
**0**	1997 /2988	66.8
**1**	1596 /2368	67.4
**2**	683 /989	69.1
**3+**	255 /404	63.1

The error rate for FRAXA and FRAXE genotypes (i.e. CGG repeat number difference >1) were 0.52% and 0.65% respectively, calculated by comparing duplicate samples. Duplicates that were not matched and samples which failed to amplify were categorised as failed ( Ennis *et al*., 2006).

### FRAXA

**Figure 1.**
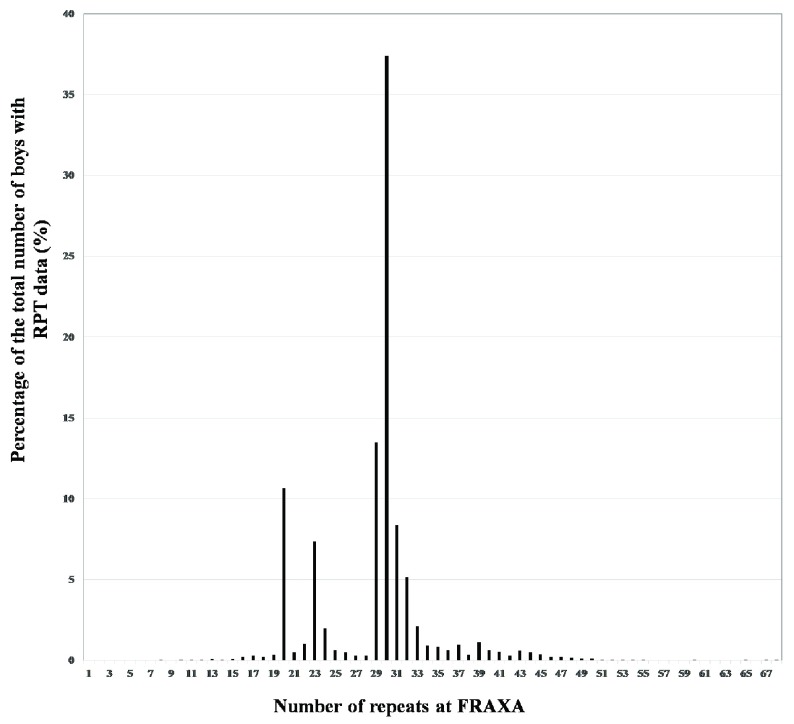
Distribution of FRAXA repeats. Graph of the distribution of number of repeats at FRAXA, for all boys for which valid number of repeats (RPT) data was obtained.

The mean number of repeats (RPTs) at FRAXA is 28.92 (S.D. 5.44), with a median of 30 and a range of 8 to 68. Using the arbitrary definitions in the literature ( Murray *et al.*, 1996), only three of the boys would be categorised as having a premutation (defined as 61-200 RPTs) and 168 as intermediary (41-60 RPTs) ( Table 2). In all, approximately 11% have an RPT >32. The frequency distribution also has a substantially high number of individuals with repeat numbers of 20 (10.67%) and 23 (7.35%) disrupting the normal distribution around the mean ( Figure 1).

**Table 2.** The frequency of boys in each class of FRAXA repeats. This is also expressed as a percentage of the number of boys for which the number of repeats (RPT) data is available.

Class of FRAXA repeat	Frequency	Percentage
Minimal (<11)	<5	0.06
Common (11-40)	4,913	96.58
Intermediate (41-60)	168	3.3
Premutation (61-200) unmethylated	<5	0.06
Total	5,087	100

### FRAXE

**Figure 2.**
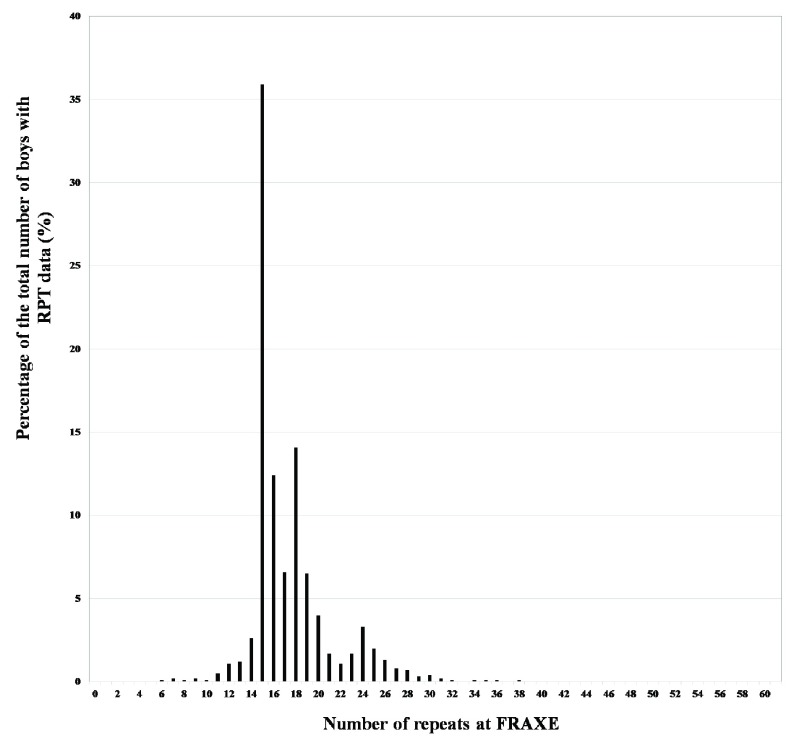
Distribution of FRAXE repeats. Graph of the distribution of number of repeats at FRAXE, for all boys for which valid number of repeats (RPT) data was obtained.

The mean number of RPTs at FRAXE is 17.41 (S.D. 3.94), with a median of 16 and a range of 0 to 61. Using the definitions in the literature ( Murray *et al*., 1996), one of the boys would be classed as having a premutation ( Table 3). There is less variation in the FRAXE RPTs compared to the FRAXA RPTs ( Figure 2), but a suggestion of a bimodal distribution. The pattern of data is unchanged across ethnicities for both FRAXE and FRAXA (5% non-white).

**Table 3.** The frequency of boys in each class of FRAXE repeats. In the third column, this is expressed as a percentage of the number of boys for which the number of repeats (RPT) data is available.

Class of FRAXE repeat	Frequency	Percentage
Minimal (<11)	43	0.85
Common (11-30)	4983	98.28
Intermediate (31-60)	43	0.85
Premutation (61-200) unmethylated	<5	0.02
Total	5070	100

## Strengths and limitations of the dataset

One of the strengths of this data is that it includes a very large number of individuals and we present the general variation across RPT sizes, which is sometimes overlooked in the literature. Additionally, given the extensive information gathered about children in the ALSPAC study, there is potential to look at the RPT size variation to ascertain if there are any correlations with other variables. This could open up interesting avenues of research into the phenotypes associated with different RPT sizes, particularly for the allele categories rarely studied in the literature so far (such as ‘minimal’ and ‘common’) and for the relatively unexpected peak seen in the FRAXA data around 20/23 RPTs ( Figure 1). Very few studies have looked at phenotypes associated with very low numbers of repeats at FRAXA or FRAXE. A few smaller studies did find association of alleles of less than 26 repeats at FRAXA with increased risk of developing fertility problems, behavioural problems and/or having children with developmental disability or psychiatric illness; this has not been replicated by larger studies and as such remains controversial ( Kraan *et al*., 2018).

There was some bias in that the boys without RPT data were more likely to have had mothers who were younger, less well educated and of lower social class ( Table 1). This needs to be taken into account in further studies, including subsequent correlations with variables from the ALSPAC data. Another limitation is that ascertaining repeat sizes of large premutation or full mutation alleles was not possible and these data are not represented in the dataset. Additionally, as discussed in the introduction the boundaries of classes of repeat numbers vary across studies, and particularly for FRAXA there is another commonly used distinction (intermediate as 41-54 repeats and premutation as 55-200 repeats). However, if we classify the boundaries this way, then the intermediate percentage only decreases a very small amount to 3.24 and premutation increases to 0.12. Although differing boundaries limits comparison across studies, we have included a full table of genotype data in Supplementary material (DOI: 10.6084/m9.figshare.11695440) such that researchers can classify whichever way they prefer.

## Data availability

### Underlying data

ALSPAC data access is through a system of managed open access. The steps below highlight how to apply for access to the data included in this paper and all other ALSPAC data. Please read the ALSPAC access policy ( http://www.bristol.ac.uk/media-library/sites/alspac/documents/researchers/data-access/ALSPAC_Access_Policy.pdf), which describes the process of accessing the data and biological samples in detail and outlines the costs associated with doing so.

1. You may also find it useful to browse our fully searchable research proposals database ( https://proposals.epi.bristol.ac.uk/), which lists all research projects that have been approved since April 2011.
2. Please submit your research proposal ( https://proposals.epi.bristol.ac.uk/) for consideration by the ALSPAC Executive Committee using the online process. You will receive a response within 10 working days to advise you whether your proposal has been approved.
  If you have any questions about accessing data, please email: alspac-data@bristol.ac.uk (data) or bbl-info@bristol.ac.uk (samples).
  The ALSPAC data management plan ( http://www.bristol.ac.uk/media-library/sites/alspac/documents/researchers/data-access/alspac-data-management-plan.pdf) describes in detail the policy regarding data sharing, which is through a system of managed open access.

### Extended data

The supplementary material for this paper is a table of the genotype data: the frequencies of numbers of repeats at FRAXA and FRAXE. This can be accessed at DOI: 10.6084/m9.figshare.11695440.
