# Supplementary material for: The FRAXA and FRAXE allele repeat size of boys from the Avon Longitudinal Study of Parents and Children (ALSPAC)
Source: Wellcome Open Res. 2020 Jan 27;4:116. Originally published 2019 Aug 2. [Version 2] doi: 10.12688/wellcomeopenres.15342.2 (PMC7096154; doi:10.12688/wellcomeopenres.15342.2)
Supplement: Peer review history

# Peer review: Reviewer response for version 2

**DOI:** 10.21956/wellcomeopenres.17209.r37691

**Published:** 2020-03-20

**Authors:** Samuel S. Chong, Timing Liu

**Affiliations:** 1 Department of Pediatrics, Yong Loo Lin School of Medicine, National University of Singapore, Singapore, Singapore; 2 Faculty of Biology, University of Cambridge, Cambridge, UK; 3 Khoo Teck Puat - National University Children's Medical Institute, National University Health System, Singapore, Singapore

We thank the authors for indicating the percentage of non-white individuals in the study, and for the table of detected allele frequencies. We understand the authors’ reluctance to re-analyze the 164 samples for whom no alleles could be detected, given that the original analyses were probably generated more than two decades ago. However, this does mean that frequencies are available only for FRAXA alleles of not more than 68 repeats, and only FRAXE alleles of 61 or less repeats. As such, the reported frequencies of normal and intermediate FRAXA and FRAXE allele spectra can be accepted without question, but the premutation allele spectra and frequencies are incomplete in this study. On a separate note, we previously wrongly assumed that the relatively high percentage of the 20 and 23 repeat FRAXA alleles was novel. It has come to our attention, however, that there have been studies reporting relatively high frequencies of 20 and/or 23 repeats in certain European groups ^1^^,^^2^.

We confirm that we have read this submission and believe that we have an appropriate level of expertise to confirm that it is of an acceptable scientific standard.

## References

[1] : Analysis of FMR1 (CGG)(n) alleles and DXS548-FRAXAC1 haplotypes in three European circumpolar populations: traces of genetic relationship with Asia. *Eur J Hum Genet*.2001;9(9) : 10.1038/sj.ejhg.5200697 724-7 10.1038/sj.ejhg.5200697 11571563

[2] : Distribution of CGG repeats and FRAXAC1/DXS548 alleles in South American populations. *Am J Med Genet*.2002;111(3) : 10.1002/ajmg.10572 243-52 10.1002/ajmg.10572 12210320

# Peer review: Reviewer response for version 2

**DOI:** 10.21956/wellcomeopenres.17209.r37690

**Published:** 2020-03-03

**Authors:** Randi J. Hagerman

**Affiliations:** 1 Medical Investigation of Neurodevelopmental Disorders (MIND) Institute, University of California, Davis, Sacramento, CA, USA

I thank the authors for adding the table of the allele sizes through the CGG repeats that they were able to detect, but I am still concerned about the 164 patients with a high end premutation or full mutation that they were not able to detect. Therefore their data cannot be used for prevalence figures for the premutation ranges or higher. If they are able to use the newer PCR techniques that can detect high end premutation carriers, then their data will be useful for prevalence studies.

I confirm that I have read this submission and believe that I have an appropriate level of expertise to confirm that it is of an acceptable scientific standard.

# Peer review: Reviewer response for version 1

**DOI:** 10.21956/wellcomeopenres.16752.r36650

**Published:** 2019-10-16

**Authors:** Samuel S. Chong, Timing Liu

**Affiliations:** 1 Department of Pediatrics, Yong Loo Lin School of Medicine, National University of Singapore, Singapore, Singapore; 2 Faculty of Biology, University of Cambridge, Cambridge, UK; 3 Khoo Teck Puat - National University Children's Medical Institute, National University Health System, Singapore, Singapore

Clark *et al. *present some nice data on the distribution of triplet repeat sizes at the FRAXA and FRAXE fragile sites in a large population sample from the ALSPAC study.

 It would be good for the authors to provide information about the break-down of the repeat size distribution by ethnic groups, given the UK population’s ethnic heterogeneity, unless all the samples are of single origin Caucasian ethnicity. This point is pertinent given that the high frequency of 20- and 23-repeat FRAXA alleles in the ALSPAC cohort has not been previously reported elsewhere.

 The authors have suggested that future studies could look at phenotypes associated with very low numbers of repeats at FRAXA or FRAXE, and certainly the 20- and 23-repeat FRAXA alleles would be interesting study candidates. Besides the repeat size, repeat structure (e.g. repeat interruptions) might also be worth a look with regard to transmission stability and cognitive phenotype variability/expressivity.

 Another point that needs addressing is the fate of the 164 samples that were excluded from further analysis due to standard PCR amplification failures. The authors acknowledged that some of these samples likely include high-level pre-mutation and full mutation samples. It is a pity that there were no follow-up analyses of these samples. Judging from the slab gel instruments used, the PCR genotyping was likely performed in the 1990s and Southern blot re-analysis then may not have been an option if DNA quantity was limited. However, the authors ought to now consider re-analysing these few samples using the newer and more powerful TP-PCR methods capable of sizing all premutations and detecting all full mutations. This would render a more complete picture of the FRAXA and FRAXE repeat size spectrum and allele distribution and frequencies in the study cohort. The paper’s title, and the statement “a dataset of repeat size frequencies at FRAXA and FRAXE of boys from the ALSPAC study”, would then also be more justified.

 Given the different repeat size classifications adopted in different studies, it would be useful if the individual genotype information be made available in the supplementary information. This will enable readers to re-plot and re-classify allele sizes according to their preferred standard.

We confirm that we have read this submission and believe that we have an appropriate level of expertise to confirm that it is of an acceptable scientific standard, however we have significant reservations, as outlined above.

# Response

**Published:** 2020-01-22

**Authors:** Rosie Clark

**Affiliations:** University of Bristol, UK

We thank the reviewer for their constructive comments. 

In response to the point regarding phenotypes associated with low numbers of repeats, while we agree that this would be worth looking at, the aim of this paper is to highlight the data that are available so that other researchers can investigate different research questions. This is what we understand to be the purpose of a Data Note paper.

Regarding the comment about PCR amplification, this is a good point but these methods are beyond our abilities and facilities currently. However, data from the ALSPAC biobank is available to researchers as outlined in our access policy. 

All other comments have been addressed by changes to the text and new supplementary material provided.

# Peer review: Reviewer response for version 1

**DOI:** 10.21956/wellcomeopenres.16752.r36130

**Published:** 2019-08-12

**Authors:** Randi J. Hagerman

**Affiliations:** 1 Medical Investigation of Neurodevelopmental Disorders (MIND) Institute, University of California, Davis, Sacramento, CA, USA

Clark *et al.* describes the alleles that were obtained from a remarkable sample, the ALSPAC study which followed the offspring of women who delivered between April 1,1991 and December 31, 1992. PCR testing was done for FRAXA and FRAXE alleles and reported here. Only boys were studied and only about 66% had successful results. The reasons for a low uptake included a lack of consent, which is understandable, but there were 164 samples where the assay did not work either because it was a high premutation or a full mutation or not enough DNA or a heterogeneous genotype with possibly 2 Xs or maternal contamination. They need to clarify the numbers that had a large premutation or a full mutation to help with the prevalence figures and if possible use Southern Blot testing or newer PCR techniques to clarify who had a full mutation. For the data that they did obtain there was a large number with a lower repeat in the FRAXA group, specifically 10.67% with 20 repeats and 7.35% with 23 repeats which is unusual compared to previous studies. It seems that the authors have missed a great opportunity to correlate these low repeat numbers with the cognitive data that seems to be available at multiple time points in the ALSPAC study. The authors should do this if possible.

 It is difficult to compare their prevalence results to previous studies because the standard definition of the premutation as 55 to 200 repeats is not used. Instead the premutation is defined as 61-200 and the intermediate is 41-60. Most other studies will use 41 to 54 repeats for the intermediate or gray zone. It would be helpful for them to define these categories in the introduction and use the standard definitions.

 Under ALSPAC sample first paragraph “enrollment” is misspelled.

 Overall this study demonstrates a high level of low CGG repeats in the FRAXA analysis but they have the opportunity to correlate this with the clinical data including cognitive testing and they need to do this.

I confirm that I have read this submission and believe that I have an appropriate level of expertise to confirm that it is of an acceptable scientific standard, however I have significant reservations, as outlined above.

# Response

**Published:** 2020-01-22

**Authors:** Rosie Clark

**Affiliations:** University of Bristol, UK

We thank the reviewer for their constructive comments. Regarding their first point about numbers of individuals with a full mutation, these methods are beyond our abilities and facilities currently, but data from the ALSPAC biobank is available to researchers for further use as outlined in our access policy.

In response to the reviewer's second point about correlating repeat numbers with cognitive data, we agree that these points would be worth looking at but the aim of this Data Note paper is to highlight the data that are available so that other researchers can investigate different research questions.

Enrolment is the English spelling so we have not corrected this. 

Other comments have been addressed as amendments to the text and supplementary material.
